# Machine Learning for Mental Health in Social Media: Bibliometric Study

**DOI:** 10.2196/24870

**Published:** 2021-03-08

**Authors:** Jina Kim, Daeun Lee, Eunil Park

**Affiliations:** 1 Department of Applied Artificial Intelligence Sungkyunkwan University Seoul Republic of Korea

**Keywords:** bibliometric analysis, machine learning, mental health, social media

## Abstract

**Background:**

Social media platforms provide an easily accessible and time-saving communication approach for individuals with mental disorders compared to face-to-face meetings with medical providers. Recently, machine learning (ML)-based mental health exploration using large-scale social media data has attracted significant attention.

**Objective:**

We aimed to provide a bibliometric analysis and discussion on research trends of ML for mental health in social media.

**Methods:**

Publications addressing social media and ML in the field of mental health were retrieved from the Scopus and Web of Science databases. We analyzed the publication distribution to measure productivity on sources, countries, institutions, authors, and research subjects, and visualized the trends in this field using a keyword co-occurrence network. The research methodologies of previous studies with high citations are also thoroughly described.

**Results:**

We obtained a total of 565 relevant papers published from 2015 to 2020. In the last 5 years, the number of publications has demonstrated continuous growth with *Lecture Notes in Computer Science* and *Journal of Medical Internet Research* as the two most productive sources based on Scopus and Web of Science records. In addition, notable methodological approaches with data resources presented in high-ranking publications were investigated.

**Conclusions:**

The results of this study highlight continuous growth in this research area. Moreover, we retrieved three main discussion points from a comprehensive overview of highly cited publications that provide new in-depth directions for both researchers and practitioners.

## Introduction

### Background

Artificial intelligence (AI) has permeated various daily sectors that are directly related to our lives [1,2]. With this trend, AI for health, which refers to applying AI to real-world health care, has become one of the most important social issues at present [3,4]. With privacy and security as the bedrock of AI-based health care, there have been many attempts to employ AI and its applications in health care services [5,6]. As a representative example, Rizwan Malik, a radiologist in the United Kingdom, adopted a unique AI-based chest X-ray system to reduce patient waiting time in the COVID-19 pandemic scenario [4]. Furthermore, Microsoft [2] has invested approximately US $20 million to aid the collaboration teams of health care professionals and data science/AI experts in COVID-19–related research.

Extensive efforts have been put forward to employ AI technologies in health care services in addressing issues related to physical health, involving several medical centers, researchers, and organizations, as well as for mental health as a rapidly growing social issues. Although mental health is a pervasive and comprehensive issue, its detection and exposure are challenging. The World Health Organization estimates that approximately 1 billion people worldwide have mental disorders [7]. Moreover, 264 million people have been globally affected by depression, a common mental disorder [8]. However, more than 75% of people in underdeveloped countries (ie, low-income countries) suffering from mental disorders do not receive any treatments [7]. Several scholars have also revealed that individuals who suffer from mental disorders tend to prefer sharing their personal information and seeking assistance to reduce their concerns through online channels rather than with medical providers such as counselors or therapists [9-11].

Considering this tendency, social media represents a supportive tool for these individuals [11], where users are allowed to generate content, share information, and communicate [12]. Many researchers have attempted to explore the large-scale user-generated content in social media by means of machine learning (ML), which is a robust data-engineering technique, to analyze hidden information and knowledge on mental health. Therefore, we provide a theoretical background of related studies in the following subsections.

### Related Review Papers

Higgins et al [13] define a systematic review as

a study that is composed of a search for scientific publications related to various topics in accordance with systematic guidelines including the search queries, the scientific databases, and the assessment criteria.

With this concept, several prior mental health studies have investigated how to utilize ML in social media datasets. For instance, Seabrook et al [14] examined a systematic approach to provide an overview of prior research that focused on depression and anxiety in social media contexts between 2005 and 2016 with 8 identified databases. To objectively evaluate and summarize the literature, each case was evaluated by three unique dimensions: how to include psychological/cognitive measures, how to use external measurements for mental health criteria, and how to collect user activities in social media. Subsequently, 70 cases were selected, examined, and reviewed for both the implications and future directions regarding the application of ML to mental health in social media.

In addition to a systematic review approach, a scoping review may be performed, which is defined as “a type of research synthesis that aims to map the literature on a particular topic or research area and provide an opportunity to identify key concepts” [15]. This implies that a scoping review provides a bird’s eye view of key concepts in specific research areas, main sources, findings, and implications. For instance, Shatte et al [16] adopted a scoping review approach including 300 papers that focused on ML and big data applications in mental health, and concluded that the majority of these papers considered depression, schizophrenia, and Alzheimer disease as their main mental illnesses. Moreover, 89% of the papers analyzed utilized supervised learning approaches such as support vector machine (SVM), naïve Bayes, or decision trees to examine their selected illness.

Chancellor et al [17] also performed a thematic discourse analysis on 55 scientific papers with the goal of predicting mental health status in social media, and demonstrated that interdisciplinary researchers have different perspectives toward users’ datasets; these perspectives were classified as “human-centered machine learning” (HCML). Based on these findings and the concept of HCML, Chancellor and De Choudhury [18] subsequently categorized a total of 75 papers with five discourses: disorder/patient, social media, scientific, data/ML, and person. Based on this categorization, a total of 75 cases in which mental health status was assessed using social media datasets within 41 conference/journal papers published from 2013 to 2018 retrieved through academic databases (eg, ACM Digital Library and Google Scholar) were reviewed with respect to data annotation methods, data collection/quality management, preprocessing procedures, feature selections, model selection, and verification.

As presented in numerous prior studies, ML and mental health in social media have gained exponential attention in both practical and academic fields. Thus, we aimed to perform a bibliometric analysis to provide an overview and recent trends of this field.

### Related Bibliometric Analyses

Bibliometric analysis is an extensive and widely used approach “to shed light on the processes of written communication and of the nature and course of development of a discipline” [19]. A bibliometric analysis thus allows researchers to understand the trends of specific research areas with several primary publications, including collaboration relations [20,21], core research themes [20], and scientific techniques [22].

Several scholars have performed bibliometric analyses on AI/ML in health care areas, including public and mental health, as well as in areas of specific mental illnesses. For example, dos Santos et al [22] performed a bibliometric analysis of data mining and ML techniques applied to public health issues based on papers published between 2009 and 2018 retrieved from three academic databases: Web of Science (WoS), Scopus, and ScienceDirect.

In the case of depression specifically, Tran et al [23] used a bibliometric approach to examine AI applications presented in publications indexed in WoS, evaluated the productivity of AI research through statistical analyses, and performed an exploratory factor analysis on the contexts of paper abstracts to present the most relevant and popular research issues. Moreover, Wang et al [24] performed a bibliometric analysis of natural language processing in various medical research areas including papers retrieved from PubMed data engines published from 1999 to 2018.

With respect to social media, several bibliometric analyses have closely evaluated relevant publications and their effects on society. As a representative example, Chen et al [20] adopted both quantitative and statistical approaches with the WoS database to detect specific events in social media within the period of 2009-2017, investigated the number of publications and degree of collaboration, and further used clustering analysis to identify the main research themes. Another bibliometric study conducted by Sa’ed et al [21] focused on social media in psychology over 12 years based on records retrieved from WoS, and identified bibliometric indicators, including international collaboration/research networks.

Based on this background, the use of ML in medical fields and social media has been extensively explored using bibliometric approaches with notable implications and future directions. Therefore, this is an appropriate time to provide more detailed observations on ML with respect to the relation of specific medical areas with social media. Specifically, we examined the trends of research using ML for mental health in social media by employing (1) a bibliometric analysis to determine the publication distributions on sources (journals or conferences), authors, institutions, countries, research subjects, and author keywords; and (2) a trend review analysis to determine the distributions of citation numbers, along with a comprehensive review of highly cited publications.

With these approaches, we aimed to identify overall research trends of this area in a quantitative manner, and to qualitatively identify the key methodologies used on diverse social media platforms. These findings can shed light on the recent trends in the field and highlight more detailed directions of future research areas.

## Methods

### Data Collection

We collected papers from two citation databases, Scopus and WoS. Scopus is one of the largest citation repositories that covers scientific journals, conference proceedings, and books. WoS stores high-quality publications evaluated by three main indices: Science Citation Index Expanded (SCIE), Social Sciences Citation Index (SSCI), or Art & Humanities Citation Index (A&HCI).

**Figure 1:**
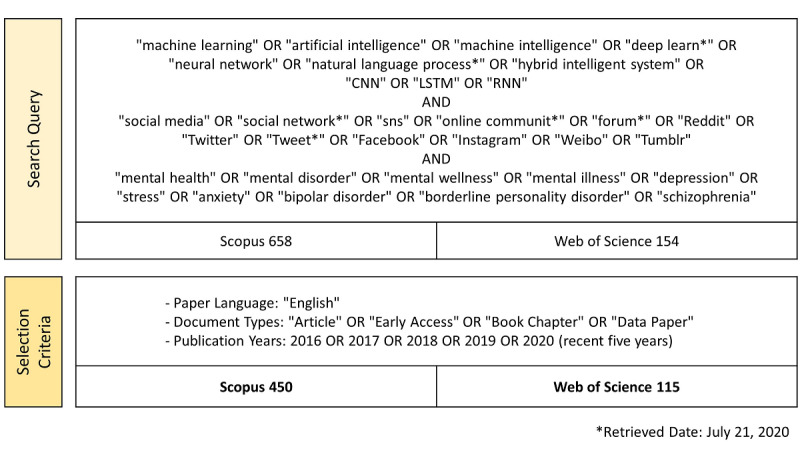
Representative data collection procedure.

Relevant publications were obtained when the terms included in the search query appeared in the title, abstract, or keywords. We defined the search query of each topic based on prior research on ML, social media [17], and mental health [25]. We excluded papers that were not written in English or were categorized as other document types (Figure 1). As a result, a total of 565 papers published from 2016 to 2020 were obtained on July 21, 2020. To cover rapidly changing trends in ML areas, we also considered the year 2020, which is still open for new issues. The complete list of included publications is provided in Multimedia Appendix 1.

### Analysis Methodologies

A bibliometric analysis includes the distribution exploration of publication and research subject, as well as citation quantities. Both the Python programming language and Microsoft Excel were employed to perform statistical analyses of the retrieved papers. We first analyzed the publication distributions of papers with several categories (eg, sources, countries, institutions, authors, and research subjects). We also performed a network analysis of frequently used keywords. Moreover, to identify the research trends in this area, we performed trend review analyses with highly cited papers covering the following topics: (i) ML techniques, (ii) specific mental illnesses, and (iii) social media.

## Results

### Publication Distribution Analysis

#### Overall Publication Trend

The continuous growth of publications from 2016 to 2020 (until July 2020) is illustrated in Table 1. In 2016, two papers were retrieved from WoS and 33 papers were retrieved from Scopus. The publication count demonstrates rapid growth in 2019 with 43 publications retrieved from WoS and 166 publications retrieved from Scopus. Considering the retrieved date (July 2020), we expect that more papers would be retrieved in the remainder of 2020 up to the present.

**Table 1:** Number of publications per year.

Year	Publication count, n (%)	
	Scopus (N=450)	Web of Science (N=115)
2016	33 (7.3)	2 (1.7)
2017	68 (15.1)	16 (13.9)
2018	88 (19.6)	21 (18.3)
2019	166 (36.9)	43 (37.4)
2020	95 (21.1)	33 (28.7)

#### Productive Publication Source

We considered several document types, including not only journal articles but also conference proceedings and book chapters. Tables 2 and 3 present the publication sources with high counts in Scopus and WoS, respectively. *Lecture Notes in Computer Science* was the most productive publication source in Scopus, followed by *CEUR Workshop Proceedings*, *Neural Computing and Applications*, and *Journal of Medical Internet Research* with more than 20 publication counts each. *Journal of Medical Internet Research* was selected as the most productive publication source in WoS with 15 publication counts, followed by *IEEE Access*.

**Table 2:** Top publication sources in Scopus (N=450).

Rank	Source	Publication count, n (%)
1	Lecture Notes in Computer Science	35 (7.8)
2	CEUR Workshop Proceedings	25 (5.6)
3	Neural Computing and Applications	22 (4.9)
4	Journal of Medical Internet Research	16 (3.6)
5	Advances in Intelligent Systems and Computing	13 (2.9)
6	ACM International Conference Proceeding Series	9 (2.0)
6	International Journal of Innovative Technology and Exploring Engineering	9 (2.0)
8	IEEE Access	7 (1.6)
9	Communications in Computer and Information Science	5 (1.1)
9	Frontiers in Psychiatry	5 (1.1)
9	International Journal of Environmental Research and Public Health	5 (1.1)

**Table 3:** Top publication sources in Web of Science (N=115).

Rank	Source	Publication count, n (%)
1	Journal of Medical Internet Research	15 (13.0)
2	IEEE Access	7 (6.1)
3	BMJ Open	3 (2.6)
3	Computers in Human Behavior	3 (2.6)
3	Frontiers in Psychology	3 (2.6)
3	IEEE Transactions on Knowledge and Data Engineering	3 (2.6)
3	International Journal of Environmental Research and Public Health	3 (2.6)
8	BMC Medical Informatics and Decision Making	2 (1.7)
8	Cyberpsychology Behavior and Social Networking	2 (1.7)
8	Journal of Information Science	2 (1.7)
8	Journal of Intelligent information Systems	2 (1.7)
8	Multimedia Tools and Applications	2 (1.7)
8	NPJ Schizophrenia	2 (1.7)
8	Scientific Reports	2 (1.7)
8	Social Science Computer Review	2 (1.7)
8	Translational Behavioral Medicine	2 (1.7)

#### Predominant Countries

More than 30 countries were identified as the predominant nations performing research in this field in Scopus (n=59) and WoS (n=39). Table 4 illustrates the top productive countries based on the number of publications. The United States was the most productive nation in both databases, followed by China and India.

**Table 4:** Top productive countries.

Rank		Country	Publication count, n (%)
**Scopus (N=450)**			
	1	United States	146 (32.4)
	2	India	66 (14.7)
	3	China	63 (14.0)
	4	United Kingdom	34 (7.6)
	5	Canada	22 (4.9)
	6	Spain	18 (4.0)
	7	Australia	17 (3.8)
	8	Germany	16 (3.6)
	9	Taiwan	14 (3.1)
	10	France	13 (2.9)
	10	Netherlands	13 (2.9)
**Web of Science (N=115)**			
	1	United States	52 (45.2)
	2	China	25 (21.7)
	3	United Kingdom	12 (10.4)
	4	Australia	11 (9.6)
	5	Spain	6 (5.2)
	6	Canada	5 (4.4)
	6	India	5 (4.4)
	6	Saudi Arabia	5 (4.4)
	7	South Korea	4 (3.5)
	7	Taiwan	4 (3.5)

#### Productive Institutions

There were 391 different institutions associated with the 565 publications. The top-ranked institutions are presented in Table 5. Harvard University in the United States emerged as the most productive institution in WoS (13 publications), whereas Tsinghua University in China was selected as the most productive organization in Scopus (21 publications).

**Table 5:** Top productive institutions.

Institution	Publication count, n (%)	
	Scopus (N=450)	Web of Science (N=115)
Tsinghua University	21 (4.7)	3 (2.6)
Georgia Institute of Technology	12 (2.7)	4 (3.5)
Harvard University	14 (3.1)	13 (11.3)
University of Pennsylvania	13 (2.9)	5 (4.4)
Chinese Academy of Sciences	14 (3.1)	6 (5.2)
Johns Hopkins University	6 (1.3)	3 (2.6)
King’s College London	6 (1.3)	—^a^
University of Toronto	6 (1.3)	3 (2.6)
National Tsing Hua University	6 (1.3)	3 (2.6)
Northwestern University	5 (1.1)	1 (0.9)
Centre National de la Recherche Scientifique	5 (1.1)	—
Vrije Universiteit Amsterdam	5 (1.1)	—
The University of Arizona	5 (1.1)	—
National University of Singapore	5 (1.1)	1 (0.9)
Deakin University	5 (1.1)	3 (2.6)
University of New South Wales	5 (1.1)	1 (0.9)
Ministry of Education China	4 (0.9)	—
Delhi Technological University	4 (0.9)	—
Cornell University	4 (0.9)	1 (0.9)
Radboud University Nijmegen	4 (0.9)	—
Russian Academy of Sciences	4 (0.9)	—
Microsoft Research	4 (0.9)	—
The University of Utah	4 (0.9)	1 (0.9)
Universidad Autónoma de Madrid	4 (0.9)	—
University of Rochester	4 (0.9)	—
University of Chinese Academy of Sciences	4 (0.9)	3 (2.6)
Université du Québec à Montréal	4 (0.9)	2 (1.7)
King Faisal University	4 (0.9)	2 (1.7)
University of Texas System	4 (0.9)	—
Asia University Taiwan	4 (0.9)	—

^a^—:no related records.

#### Predominant Authors

The top 20 researchers contributing to the field are listed in Table 6 based on their number of publications. Twelve researchers are affiliated to US-based organizations and five belong to Chinese institutions. The institutions of productive authors include not only several academic institutions but also some well-known hospitals such as Zucker Hillside Hospital. The most productive researcher was Professor Munmun De Choudhury, affiliated with Georgia Institute of Technology (15 publications), followed by Professor Sharath Chandra Guntuku from the University of Pennsylvania.‬‬‬‬‬‬‬‬‬‬‬‬

**Table 6:** Top 20 productive authors.

Author	Institution	Country	Publication count, n (%)	
			Scopus (N=450)	Web of Science (N=115)
M De Choudhury	Georgia Institute of Technology	United States	11 (2.4)	4 (3.5)
SC Guntuku	University of Pennsylvania	United States	5 (1.1)	4 (3.5)
HF Ahmad	King Faisal University	Saudi Arabia	4 (0.9)	3 (2.6)
SK Ernala	Georgia Institute of Technology	United States	4 (0.9)	3 (2.6)
LH Ungar	University of Pennsylvania	United States	4 (0.9)	3 (2.6)
T Nguyen	University of Pennsylvania	United States	5 (1.1)	2 (1.7)
S Venkatesh	University of Maryland	United States	5 (1.1)	2 (1.7)
ML Birnbaum	Zucker Hillside Hospital	United States	4 (0.9)	2 (1.7)
M Conway	University of Utah	United States	4 (0.9)	2 (1.7)
L Feng	Tsinghua University	China	4 (0.9)	2 (1.7)
D Phung	Deakin University	Australia	4 (0.9)	2 (1.7)
J Jia	Tsinghua University	China	6 (1.3)	—^a^
T Zhu	Chinese Academy of Sciences	China	6 (1.3)	—
RM Merchant	University of Pennsylvania	United States	2 (0.4)	3 (2.6)
H Christensen	University of New South Wales	Australia	3 (0.7)	2 (1.7)
JM Kane	Zucker Hillside Hospital	United States	3 (0.7)	2 (1.7)
Q Li	Tsinghua University	China	3 (0.7)	2 (1.7)
AF Rizvi	Zucker Hillside Hospital	United States	3 (0.7)	2 (1.7)
CY Shen	National Tsing Hua University	China	3 (0.7)	2 (1.7)
L Ungar	University of Pennsylvania	United States	3 (0.7)	2 (1.7)

^a^—: no related records.

#### Productive Research Subjects

**Figure 2:**
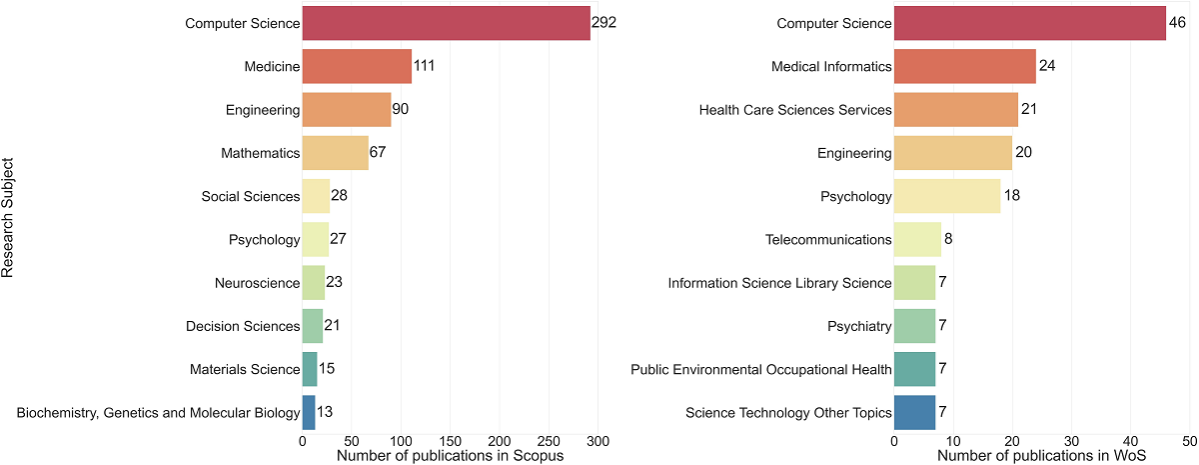
Publication count of top 10 research subjects.

The top 10 research subjects of each citation database are given in Figure 2. Among them, computer science was the most pivotal research subject in both databases (46, 40% in WoS; 292, 65% in Scopus). In Scopus, medicine (111, 25%), and engineering and mathematics (90, 20%) accounted for more than 10% of the total publications. In WoS, medical informatics (24, 21%), health care sciences services, engineering, and psychology constituted more than 10% of total publications.

#### Author Keyword Co-occurrence

**Figure 3:**
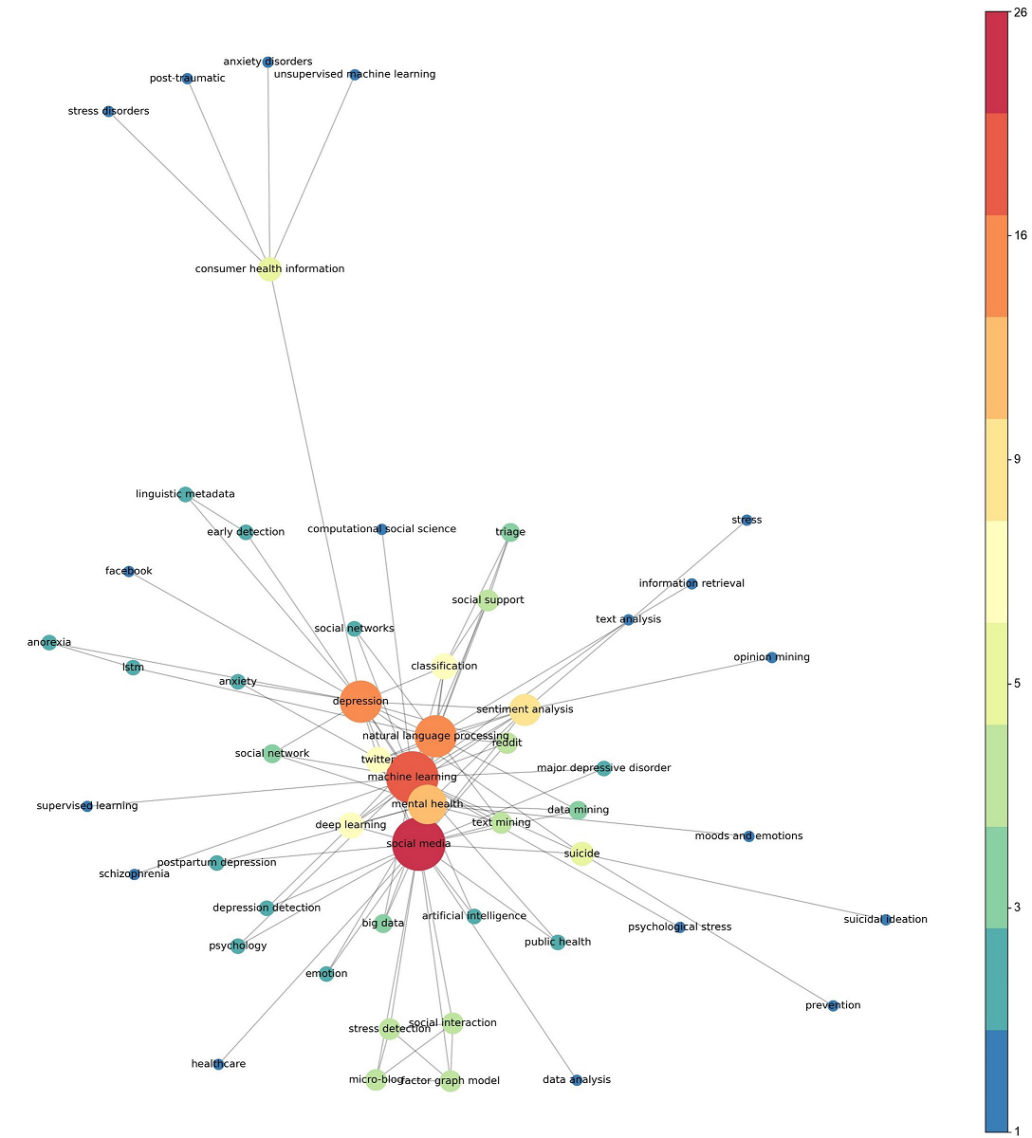
Keyword co-occurrence network graph; the color map on the right side represents the degree centrality.

Along with research subjects, we investigated the keywords derived by the authors, which represent the main research topics of the publications [26]. The keyword co-occurrence is visualized using a network graph (Figure 3), which is a well-known bibliometric methodology, in which each node is a keyword, while an edge between two nodes indicates the co-occurrence of two words. After building a network graph, we excluded edges with less than 3 co-occurrences.

Note that the keywords with a high frequency reflect the scope of the research area, which includes social media, ML, natural language processing, and mental health. In particular, in the case of mental health–related words, depression was the most frequently presented keyword, followed by suicide, consumer health information, social support, and stress detection. The main research methodologies of papers included natural language processing, sentiment analysis, classification, text mining, and stress detection in the ML field. Twitter and Reddit were identified as the most widely investigated social media platforms in this area.

As there are several approaches of text analysis using natural language processing techniques in this field, we believe that future studies may provide hybrid approaches using both textual- and visual-based data collected from several types of social media data. In addition, validating ML models trained on mental health–related social media data in clinical settings needs to be further investigated.

### Overview of Highly Cited Publications

#### Publication Citation Quantities

The annual number of citations is presented in Table 7. Along with publication distributions, the annual number of citations has been consistently increasing. Up to July 21, 2020, more than 900 and 400 annual citations were recorded in Scopus and WoS, respectively.

**Table 7:** Number of citations per year.

Year	Citation count, n	
	Scopus	Web of Science
2016	14	1
2017	112	32
2018	349	120
2019	1000	341
2020	938	420

#### Comprehensive Analysis of Highly Cited Papers

We evaluated the overall academic output through bibliometric analysis. Due to the lack of observations in the content of the publications, as mentioned in previous studies [20,27], we extensively observed and reviewed the top five most highly cited papers per year to identify the comprehensive research methodologies in the field (Table 8). After excluding 7 duplicated papers, 39 papers were selected. Subsequently, a two-round filtering procedure was performed to determine whether a specific paper meets the following criteria: (i) addressing specific mental illness, (ii) using an ML technique, and (iii) utilizing datasets of social media. There were 15 papers that met these criteria. Subsequently, three experts in ML, medical services, and computer science, respectively, participated in the second-round filtering procedure. Following this, 10 papers that satisfied these criteria were selected.

**Table 8:** Overview of the top 5 most cited papers by year.

Year	Top-cited papers (N=39), n		Reviewed papers (N=10), n	
	Article	Conference paper	Article	Conference paper
2016	5	2	2	1
2017	6	0	3	0
2018	9	0	2	0
2019	8	2	1	0
2020	6	1	1	0

An overview and the research methodologies of the 10 highly cited publications are listed in Table 9, which are categorized according to the data source: Twitter (n=4 publications), Instagram (n=3), Facebook (n=3), Reddit (n=3), Weibo (n=2), and other online community (n=1).

**Table 9:** Summary of research methodologies employed in highly cited publications.

Data Source	Reference	Mental Health	Data Description	Machine learning				
				Model	Feature	Output	Annotation	Results
Twitter	Budenz et al [28]	Mental illness, bipolar disorder	1,270,902 tweets including bipolar or mental health-related words	Logistic regression	Term frequency-inverse document frequency	Related to mental illness or bipolar disorder	Manually annotated 2047 tweets with topic, stigma, and social support messaging	10-fold cross validation (AUC^a^=0.83)
Twitter	Du et al [29]	Suicide	1,962,766 tweets including 21 suicide-related keywords/phrases	CNN^b^, SVM^c^, extra trees, random forest, logistic regression, Bi-LSTM^d^	One-hot-vector mapped to pretrained GloVe Twitter embedding	Related to suicide	Manually annotated 3263 tweets and trained classifier to select 3000 additional suicide-related tweets	Accuracy 0.74, recall 0.96, precision 0.78, F1 0.83
Facebook, Twitter	Guntuku et al [30]	Psychological stress	601 users’ Facebook and Twitter posts	Linear regression with several regularization methods (eg, ridge, elastic-net, LASSO^e^ and L2 penalized SVMs)	LIWC^f^, latent Dirichlet allocation topic modeling, stress lexicon, user engagement	Stress	Qualtrics survey; fill out the demographic questions and Cohen 10-item Stress Scale	5-fold cross-validation (Pearson *r*=0.24); trained on Facebook and Twitter, tested on Twitter
Facebook, Instagram	Shuai et al [31]	Social network mental disorders (eg, cyber-relationship addiction, information overload, and net compulsion)	3126 users' Instagram and Facebook data	Decision tree learning, SVM, logistic regression, DTSVM^g^, SNMDD^h^ (newly proposed model; tensor technique for deriving latent features)	Social interaction, personal profile, duration	Social network mental disorders	MTurk survey - fill out the standard social network mental disorder questionnaires; professional psychiatrists labeled users having a social network mental disorder	5-fold cross validation (accuracy0.78 for Instagram and 0.83 for Facebook)
Instagram	Reece and Danforth [32]	Depression	43,950 users’ Instagram images	Random forest classifier	Number of comments and likes, number of faces in photograph, 3 color properties (hue, saturation, value)	Depression	MTurk survey; Center for Epidemiologic Studies Depression Scale to measure depression level	Recall: 0.697; precision: 0.604; F1: 0.647
Weibo	Lin et al [33]	Stress	1 billion Weibo posts	SVM, softmax regression, gradient-boosted decision tree, LASSO-MTL^i^, L2-MTL^j^, cASO-MTL^k^	CNN features or word vector representations with hand-crafted features	12 stressor events (eg, marriage, financial, illness, school), 6 stressor subjects	30 volunteers manually annotated the stressor events and subjects	10-fold cross validation (F1>0.80)
Weibo	Cheng et al [34]	Suicide risk, depression, anxiety, stress	974 participants’ Weibo posts, suicide probability, Weibo suicide communication (WSC), depression, anxiety, and stress.	SVM	Simplified Chinese-LIWC	Suicide risk, emotional distress (depression, anxiety, stress), WSC	Survey and psychological test tools (ie, Suicide Probability Scale, Depression Anxiety Stress Scales-21)	leave-one-out cross-validation: suicide probability (AUC=0.61, *P*=.04), severe anxiety (AUC=0.75, *P*<.001)
Reddit	Gkotsis et al [35]	Bipolar, schizophrenia, anxiety, depression, self-harm, suicide watch, addiction, crippling alcoholism, opiates, autism	1,014,660 posts	CNN, FF^l^, linear regression, SVM	Word vector representation (16 vector size)	Mental health	N/A^m^	Accuracy: 91.8% (binary classification task), 79.8% (multiclass classification task)
Facebook, Twitter, Instagram, Reddit	Coppersmith et al [36]	Suicide risk	197,615 posts from 418 users	LSTM with attention	One-hot-vector mapped to pretrained GloVe	Suicide risk	Examining public self-stated data and using data donated through OurDataHelps.org	10-fold cross validation (AUC=0.94)
Online Community - Live Journal	Saha et al [37]	Mental health	620,060 posts from 78,647 users	MTL	Linguistic features of LIWC; topics by LDA^n^	Mental health subreddit (eg, Abuse, Anorexia, Anxiety, Bipolar disorder, Cutting, Death, Drugs, Eating disorders, Insomnia, Pain, Self-injury, and Suicide)	N/A	AUC=0.94 with the community on eating disorders

^a^AUC: area under the curve.

^b^CNN: convolutional neural network.

^c^SVM: support vector machine.

^d^Bi-LSTM: bidirectional long short-term memory.

^e^LASSO: least absolute shrinkage and selection operator.

^f^LIWC: Linguistic Inquiry and Word Count.

^g^DTSVM: decision tree support vector machine.

^h^SNMDD: social network mental disorder detection.

^i^MTL: multitask learning.

^j^l2-MTL: multitask learning considering l2 loss.

^k^cASO-MTL: clustered alternating structure optimization multitask learning.

^l^FF: feed-forward.

^m^N/A: not applicable.

^n^LDA: latent Dirichlet allocation.

In the case of Twitter, Budenz et al [28] analyzed 1,270,902 tweets by searching bipolar and mental health terms. Before using a logistic regression analysis to classify whether a specific tweet was asking for any help or included terms associated with mental illness stigma, they repeatedly performed a series of sentiment analyses on 2047 randomly sampled tweets. The results obtained through 10-fold cross-validation procedures showed an average area under the curve (AUC) of 0.83 when the term-frequency inverses document frequency weighted vector was employed as the input source.

Twitter tweets were also used to predict an association with psychological stressors, as one of the major causes of suicide. To prevent suicidal behaviors [29], the authors retrieved 1,962,766 tweets based on 21 suicide-related keywords, manually annotated labels of a subset of 3263 tweets, and labeled the other 3000 tweets based on ML classifiers. A Twitter corpus–pretrained GloVe vector was employed to convert each token into a vector as an input of the convolutional neural network (CNN). The CNN model achieved an F1-score of 83%, which outperformed SVM, extra trees, and other ML algorithms.

In addition, stress level, as one of the pervasive causes of mental health conditions, was predicted on social media platforms, including Twitter and Facebook [30]. Linguistic characteristics were extracted from a total of 601 users’ social media posts using the Linguistic Inquiry and Word Count (LIWC) tool, latent Dirichlet allocation (LDA), and stress lexicon. To predict stress, the authors applied linear regression with regularization methods, and validated the model performance based on sociodemographic variables (eg age, gender, race, income, and education) and social media language using the Pearson correlation coefficient (*r*). The content analysis indicated significant differences in language expressions among social media platforms.

Shuai et al [31] collected posts from both Facebook and Instagram to detect social network mental disorders (SNMD), which include several side effects such as cyber-relationship addiction or net compulsion. They developed and employed SNMD questionnaire items to classify each participant into specific types of SNMD. Among more than 3100 participants, 389 respondents were regarded as having SNMD. Social interaction, personal profile, and duration extracted by each participant’s social activity logs were employed as input features of ML models. The proposed model, which was organized by new tensor techniques and latent features, achieved more than 83% accuracy in identifying whether a specific user has SNMD.

One of the notable approaches in this area is a visual-oriented approach. Reece and Danforth [32] employed 43,950 images from 166 Instagram users to detect posts related to depression. Based on the results of the Center for Epidemiologic Studies Depression Scale questionnaire (CES-D), a total of 71 users revealed that they experienced depression. Moreover, both Instagram usernames and history were collected from crowd workers who responded to the CES-D. They extracted metadata (eg, the number of comments, “likes”), color properties (eg, hue, saturation, value), and the total number of faces from the collected photographs to investigate whether users suffer from depression.

Based on the guidelines of De Choudhury et al [38], Reece and Danforth decided to integrate the users’ recent posts presented on a specific (single) day rather than using their entire posts. Through a random forest classifier, they achieved a relatively high recall score of identifying the target class at 70% in 100 observations. The results indicated that the photos posted by depressed users were more likely to be bluer, grayer, and darker, and to receive fewer likes. However, as a limitation of the study, they pointed out that depression is a form of general clinical status, indicating a need for fine-tuning the questionnaires for specific diagnosis.

Lin et al [33] collected approximately 1 billion tweets from the Chinese social media platform Weibo, and proposed ML multitask models to detect both stressor events and six subjects. The event was categorized into 12 different labels, including marriage, financial, illness, and school. Each tweet was first labeled as stress-related. The tweets were categorized into one of the stressor events and subject categories by 30 volunteers. The performance of classifying a stressor event or subject was represented with various classifiers such as SVM, softmax regression, and gradient decision. The model performance was not clearly presented; however, it was stated that the F1-score reached over 80% in the event detection task.

A mental illness is often accompanied by another mental illness as a so-called “comorbidity,” which refers to the simultaneous presence of one or more mental or physical disorders. From this viewpoint, Saha et al [37] developed a joint learning model, which was generated by multitask learning to simultaneously identify co-occurring social media communities related to mental health with consideration of the correlation between the communities. Based on 620,060 posts of 78,647 users in 247 online communities, 12 major mental health–related topics were employed in the categorization standards from “Live Journal” (eg, Abuse, Anorexia, Anxiety, Bipolar disorder, etc). Using these data, two features were extracted as inputs: language style (from the LIWC) and topics (from the results of LDA). In general, the proposed model outperformed single-task learning [39] and multitask learning [40] for 9 out of 12 and 8 out of 12 categories, respectively. Moreover, the model achieved an AUC of 0.94 with the community on eating disorders.

Cheng et al [34] utilized Weibo data to assess the levels of suicide risk and emotional distress such as depression, anxiety, and stress. For this purpose, the researchers completed an internet survey and gathered 974 respondents’ Weibo posts, the scores of mental health (ie, suicide probability, depression, anxiety, and stress) through psychological investigation tools, and Weibo Suicide Communication (WSC), which examined whether respondents had told others that they wanted to commit suicide through Weibo over the past 12 months. SVM was applied for a binary classification of five suicide risk factors (suicide probability, depression, anxiety, stress, and WSC), including 72 linguistic features of Simplified Chinese-LIWC from the respondents’ Weibo posts as independent variables. The model efficiently classified the respondents having a high suicide probability (AUC=0.61, *P*=.04) and severe anxiety (AUC=0.75, *P*<.001) among those who had WSC with leave-one-out cross-validation procedures.

Gkotsis et al [35] collected user-oriented data from the Reddit community to develop deep-learning models for classifying posts according to mental disorder topics. After an expert panel made a decision on whether a specific post contains mental health–related issues, they collected 10,146,60 posts and extracted 11 mental disorder themes, including a nonmental health conditions. To classify whether a specific post belongs to one of the mental health topics, they employed a CNN model with two parallel classification approaches: binary and multiclass classifications. The word vectors of each token extracted from post texts were used as input. The results of the model demonstrated that the CNN classifier showed 91.8% and 79.8% accuracy in binary and multiclass classification tasks, respectively.

Early estimation of a person’s suicide risk is also an important issue in our society. In accordance with this point, Coppersmith et al [36] employed social media data to predict the level of suicide risk using a long short-term memory (LSTM) model. Two different datasets were collected: one from donated data through OurDataHelps.org, which included social media data of suicide victims (eg, Facebook, Instagram, Twitter), and the other from Harman Dredze [41], which provided Twitter data from users who mentioned their past suicide attempts in tweets. A total of 197,615 posts from 418 users were obtained. Based on the pretrained GloVe embeddings to feed sequences of word vectors into the bidirectional LSTM model, an AUC of 0.94 was achieved through 10-fold cross-validation procedures.

## Discussion

This study involved a bibliometric analysis on the publications related to ML and mental health in social media from 2015 to 2020 with two citation databases, WoS and Scopus, as well as a trend review analysis. Although several prior studies have investigated mental illness, the majority of these studies employed both clinical and physical health care approaches. Along with these studies, social media is considered as one of the most important spaces for effectively and efficiently addressing individuals’ mental health issues [42]. Furthermore, with rapidly improved ML and big data techniques, both the significance and importance of employing social media and online communications are being consistently emphasized.

Rapidly and consistently increasing publication and citation numbers indicate that there is growing attention and interest in this research area. Among several publication venues, *Lecture Notes in Computer Science* (Scopus) and *Journal of Medical Internet Research* (WoS) were the most productive publication sources in this field. Moreover, Harvard University and Georgia Institute of Technology in the United States, and Tsinghua University and the Chinese Academy of Sciences in China were listed as the most vigorous institutes. For individual researchers, Professor Munmun De Choudhury from Georgia Institute of Technology emerged as the most productive and well-known researcher in this field, with 15 publications to her credit. Regarding publications, using social media data in predicting depression was Prof De Choudhury’s first step in this area [38], which allowed her affiliated nation (United States) to be the most productive country in this field. Computer science, medicine, and medical informatics were identified as the core research subjects, along with several other related subjects such as psychology, social science, and neuroscience. This suggests that this research area tends to require integrated or multidisciplinary approaches for gaining a better understanding of each research topic. In addition, the keyword co-occurrence network graph highlighted the representative ML techniques and social media for this multidisciplinary area.

Subsequently, we conducted a trend analysis review on highly cited articles, and notable research trends were identified. The highly cited articles tended to employ user-generated content in diverse forms, including text, images, and other metadata, for specific mental disorders. Because no ground truth labels exist for users who have mental disorders, the majority of studies adopted a crowdsourcing survey with a medical-oriented approach and consideration of the participants’ agreements in using their social media accounts [30-32,34]. Moreover, several scholars have employed user-oriented features, including users’ demographic profiles and activity logs, in social media (eg, comments, likes) to arrive at both academic and practical contributions [30,31].

Based on the employed approaches with several highly cited articles, three main implications for discussion can be derived. First, the majority of the articles stated privacy and ethical issues as key considerations in using ML for specific mental illness in social media [23,30,32,36]. Although they met both research ethical guidelines and participants’ agreements in using their social media data, there were notable adverse reactions from several participants in sharing their social media information [32]. Moreover, compared to other issues in this area, both privacy and ethical issues are considered to be real issues requiring more academic and practical work [23]. Thus, researchers must make efforts to effectively consider these threats, which can negatively affect data providers and leave room for abusing ML techniques. Second, because there is potential for misclassified ground truth data, there should be more detailed and systematic examinations in building the early stage of datasets [28,29,34,36]. Third, because users’ expressions on social media can consistently change over time, time-oriented approaches with differing perspectives toward users’ activities and expressions should be considered in providing a better understanding in this area [32].

Considering these discussion points, a few limitations of this study remain. Although we employed WoS and Scopus as our subjects, which are both widely used academic databases globally, there can be other medical-oriented databases that may provide more significant academic and practical information. Moreover, considering more recent and applicable statistical or natural language processing techniques (such as exploratory factor analysis [23] or topic modeling [20,23]), future research should aim at obtaining deeper and comprehensive knowledge with more creative and significant approaches through various data sources of each article.

With the increase of AI applications in real-word health care settings [1], there have been numerous attempts to overcome the limitations of offline consultations such as wearable fitness trackers [43], mobile health apps [44], and conversational agents for patients’ mental health and wellness [1,45]. Moreover, since the use of social media has been widely adopted in health care [46], we believe that our analysis may trigger all stakeholders to further consider how to employ ML approaches toward mental health in social media. In addition, when applying social media data to clinical settings, there is a need to address different characteristics of social media platforms by utilizing the substantial research background.

## Appendix

Multimedia Appendix 1 Complete publication lists.

## Abbreviations

AI: artificial intelligence
AUC: area under the curve
CES-D: Center for Epidemiologic Studies Depression Scale
CNN: convolutional neural network
HCML: human-centered machine learning
LDA: latent Dirichlet allocation
LIWC: Linguistic Inquiry and Word Count
LSTM: long short-term memory
ML: machine learning
SNMD: social network mental disorders
SVM: support vector machine
WoS: Web of Science
WSC: Weibo Suicide Communication
